# A Single-Dose Intramuscular Nanoparticle Vaccine With or Without Prior Intrauterine Priming Triggers Specific Uterine and Colostral Mucosal Antibodies and Systemic Immunity in Gilts but Not Passive Protection for Suckling Piglets

**DOI:** 10.3389/fvets.2022.931232

**Published:** 2022-08-03

**Authors:** Pooja Choudhary, Amir Khajavinia, Ramin Mohammadi, Siew Hon Ng, Nathalie Bérubé, Damayanthi Yalamati, Azita Haddadi, Heather L. Wilson

**Affiliations:** ^1^Vaccine and Infectious Disease Organization, University of Saskatchewan, Saskatoon, SK, Canada; ^2^Division of Pharmacy, College of Pharmacy and Nutrition, University of Saskatchewan, Saskatoon, SK, Canada; ^3^Alberta Research Chemicals Inc., Edmonton, AB, Canada; ^4^Department of Veterinary Microbiology, Western College of Veterinary Medicine, University of Saskatchewan, Saskatoon, SK, Canada; ^5^Vaccinology and Immunotherapeutics Program at the School of Public Health, University of Saskatchewan, Saskatoon, SK, Canada

**Keywords:** pigs, vaccine, intrauterine, intramuscular, nanoparticle, adjuvants

## Abstract

An effective single-dose vaccine that protects the dam and her suckling offspring against infectious disease would be widely beneficial to livestock animals. We assessed whether a single-dose intramuscular (i.m.) porcine epidemic diarrhea virus (PEDV) vaccine administered to the gilt 30 days post-breeding could generate mucosal and systemic immunity and sufficient colostral and mature milk antibodies to protect suckling piglets against infectious challenge. The vaccine was comprised of polymeric poly-(lactide-co-glycolide) (PGLA)-nanoparticle (NP) encapsulating recombinant PEDV spike protein 1 (PEDVS1) associated with ARC4 and ARC7 adjuvants, a muramyl dipeptide analog and a monophosphoryl lipid A (MPLA) analog, respectively (NP-PEDVS1). To establish whether prior mucosal exposure could augment the i.m. immune response and/or contribute to mucosal tolerance, gilts were immunized with the NP-PEDVS1 vaccine *via* the intrauterine route at breeding, followed by the i.m. vaccine 30 days later. Archived colostrum from gilts that were challenged with low-dose PEDV plus alum was used as positive reference samples for neutralizing antibodies and passive protection. On day 100 of gestation (70 days post i.m. immunization), both vaccinated groups showed significant PEDVS1-specific IgG and IgA in the serum, as well as in uterine tissue collected on the day of euthanasia. Anti-PEDVS1 colostral IgG antibody titers collected at farrowing were significantly higher relative to the negative control gilts indicating that the NP vaccine was effective in contributing to the colostral antibodies. The PEDVS1-specific colostral IgA and anti-PEDVS1 IgG and IgA antibodies in the mature milk collected 6 days after farrowing were low for both vaccinated groups. No statistical differences between the vaccinated groups were observed, suggesting that the i.u. priming vaccine did not induce mucosal tolerance. Piglets born to either group of vaccinated gilts did not receive sufficient neutralizing antibodies to protect them against infectious PEDV at 3 days of age. In summary, a single i.m. NP vaccine administered 30 days after breeding and a joint i.u./i.m. vaccine administered at breeding and 30 days post-breeding induced significant anti-PEDVS1 immunity in systemic and mucosal sites but did not provide passive protection in suckling offspring.

## Highlights

- Single-dose intramuscular vaccine alone or an intrauterine vaccine followed by an intramuscular vaccine can promote significant antigen-specific mucosal (uterine and colostral) and systemic antibodies, but it induced low-level colostral neutralizing antibodies.
- With this dose and formulation, an intrauterine NP vaccine did not act as a priming vaccine to an intramuscular booster nor did it induce tolerance.
- Neither vaccine was sufficient to promote protective suckling piglets against infectious PEDV.

## Introduction

The swine industry values high-reproductive performance by gilts and sows, as well as high-piglet survival and growth rates. Porcine Epidemic Diarrhea Virus (PEDV) affects all ages of animals, but it kills up to 90–100% of infected piglets within the first few days after birth if they do not receive passive immunity from their dams (1, 2). It is estimated that the net annual decrease for the U.S. economic welfare from PEDV summed across all ages of pig ranges from $900 million to $1.8 billion (3). The timely and effective immunization of gilts/sows to trigger passive protection for piglets is highly sought to improve swine health, protect against neonatal infectious diseases, and maintain a cost-effective industry.

Subunit vaccines are extremely safe options for livestock because they cannot revert to a pathogenic form. However, because subunit antigens are highly purified, they tend to be poorly immunogenic and must be formulated with adjuvants to induce strong immunity (4). We used the S1 portion of the PEDV spike protein that is essential for cellular entry, and three intramuscular immunizations with a soluble vaccine formulation in gilts in the 3rd trimester contributed to the passive protection to suckling piglets (5). Combination adjuvants can fine-tune and selectively direct the type of immune response or augment the magnitude of the immune response. Muramyl dipeptide (MDP) is a component in the bacterial cell wall component peptidoglycan. In eukaryotic cells, MDP is detected by NOD2, a cytoplasmic receptor belonging to the innate immune system (6). MDP has been shown to induce immune responses by increasing IFNγ and other cytokine production (7), stimulating the differentiation and proliferation of lymphocytes (8), and it has been shown to influence immune responses with other TLR ligands (9). ARC4 adjuvant is an MDP derivative (10). ARC7 is a glycolipid, a TLR4 ligand, and a monophosphoryl lipid A (MPLA) analog. MPLA is a detoxified derivative of LPS that has an immunomodulatory impact on the innate and adaptive immune system and has been used as a vaccine adjuvant in humans (11). Finally, poly(lactic-co-glycolic acid) (PLGA) nanoparticles (NPs) have been shown as potential delivery vehicles for immunomodulators (12–16), proteins, and peptides (12, 15, 16). Our research showed the subcutaneous immunization of mice with a polymeric NP vaccine with protein antigen plus ARC4/7 formulated poly-(lactide-co-glycolide) (PGLA) NPs triggered robust antigen-specific IFNγ and IL-17A production (10). Others have shown the MPLA combined with *Streptococcus suis* proteins provides immunological protection in pigs (17), and that the pig innate immune response was induced in response to MDP administration (18), so it is reasonable to assess whether these adjuvants will trigger an innate immune response in pigs. Herein, we investigate whether these adjuvants formulated with recombinant PEDV spike protein 1 (PEDVS1) protein as part of a PLGA NP can promote robust humoral and cell-mediated immunity (CMI) in pigs, even after a single i.m. dose.

Using a single-dose vaccine that promotes protective immunity against infectious disease would significantly benefit the pig industry. Moreover, formulating vaccines so that they can be administered *via* the intramuscular (i.m.) route yet trigger uterine and systemic immunity would help protect pig reproductive health (19). Because the majority of commercial pigs are bred by artificial insemination (AI) (20), current husbandry practices allow routine access to the uterus during each reproductive cycle. Our previous research showed that rabbits administered a single i.m. subunit vaccine triggered very high systemic and mucosal (i.e., lungs, vaginal, and mucosal) immune responses to the vaccine antigen, although we did not judge protection against the disease (21). Our work has also shown that repeated administration of an intrauterine vaccine at breeding triggered robust mucosal and systemic immunity in gilts and partial protection of suckling piglets against PEDV when they were infected 3 days after birth (22). Our primary objective is to assess the effects of a single-dose i.m. NP vaccine formulated with two adjuvants and recombinant PEDVS1 protein on the gilt local and systemic humoral and cell-mediated immune responses. An important secondary objective is to determine if sufficient maternal neutralizing antibodies were transferred to suckling piglets to protect against disease. Our tertiary objective was to discern whether prior i.u. immunization impacted the response triggered by an i.m. vaccine administered 30 days later or whether initial i.u. exposure induced mucosal tolerance.

## Materials and Methods

### Production of Recombinant Antigens

Recombinant (r) PEDVS1 [amino acids 21–734 of PEDV spike protein (accession number AG058924) with carboxyl GSGSG(H)12 added] was expressed in the human embryonic kidney cells then affinity-purified as previously described (5) (same batch used herein).

### Vaccine Formulation and Characterization

ARC4 is a lapidated muramyl dipeptide (LMDP), and MDP is known for activating the NOD2 receptor in the immune stimulation mechanism. Similarly, ARC7 is a synthetic monophosphoryl lipid A (MPLA). MPLA is the smallest active component derived from lipid A of various bacteria and has been widely studied for its activation of the TLR4 receptor. ARC4 and ARC7 were synthesized and purified by the Alberta Research Chemicals Inc. (Edmonton, AB, Canada) through proprietary means and their structures are shown in Table 1. PLGA NPs were prepared by the emulsification solvent evaporation method, as mentioned previously (23). Briefly, rPEDVS1/PBS solution (10%) was emulsified (w/o) in PLGA/chloroform solution (25%) containing 7-Acyl in chloroform:methanol (2%) or ARC4 in chloroform:methanol (2%). The resulting mixtures were further emulsified in 5% of PVA to form a secondary emulsion (w/o/w), followed by stirring for 3 h to evaporate the solvents. The NPs were then collected by ultracentrifugation. We performed ultracentrifugation of the NPs at 15 min/15,000 rpm and 20 min at 18,000 rpm using a J2-21 Ultracentrifuge (Beckman, USA). The size distribution was determined to be 477.7 ± 4.2 nm when PEDVS1 was included and 212.3 ± 4.2 nm when the PLGA NPs did not include the antigen or adjuvant. Size distribution was determined using Malvern Zetasizer (Nano series, Montreal, Canada). The Zeta potential was quantified using Malvern Zetasizer (Nano series, Montreal, Canada), and it was shown to be −20.4 ± 1.2 when PEDVS1 was included and −11.6 ± 1.7 when the NPs did not include the antigen. In the final step, the NPs were freeze-dried and stored at −20°C for further use. To prepare the vaccine, certain amounts of NPs were mixed to obtain the required dose for immunization.

**Table 1:** Schematic of ARC4 and ARC7 and targets.

**Name**	**Structure**	**Target**
ARC4	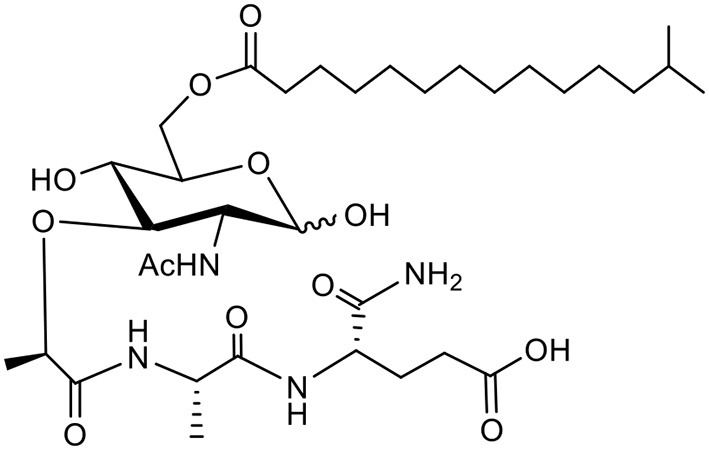	NOD2
ARC7	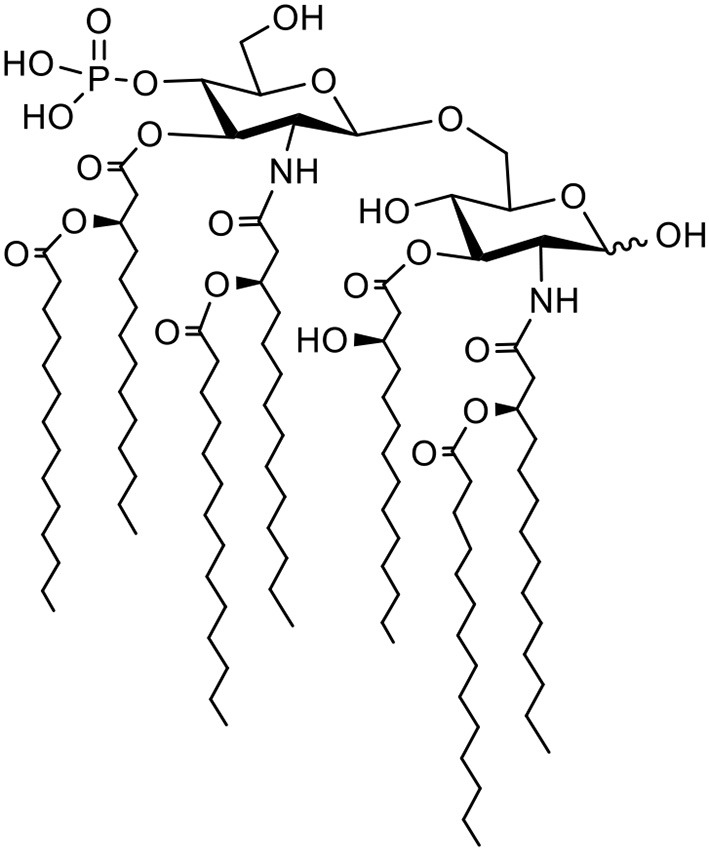	TLR4

### Evaluation of the Loading Efficiency

The amounts of adjuvants encapsulated in the PLGA NPs were determined by LC-MS/MS using a pre-column guard. For ARC7 encapsulation, a previously published method from our group was applied (24). To analyze ARC4 encapsulation, a high-performance liquid chromatography (HPLC) system was interfaced with the mass spectrometer. The Applied Biosystems/MDS Sciex Analyst software (Version 1.6.0) was used for system control and quantification. A sample volume of 5 μL was injected using the 1,200 Agilent autoinjector set to 4°C and was delivered with an isocratic mobile phase consisting of methanol (0.1% formic acid) at a flow rate of 200 μl/min for a run-time of 2 min. Mass spectrometry showed that 1.79 ± 0.18 μg per 1 mg NP or ARC7 and 335 ± 0.10 ng per 1 mg NP of ARC4 were loaded into the NPs.

Loaded PEDVS1 in NPs was extracted from the NP formulations and quantified using a BCA assay, according to our previous studies (15). The BCA analysis showed that in each mg of NP, we loaded 2.918 μg PEDVS1. For the first vaccination, we administered 180 μg of PEDVS1 per 2 ml, and for the second dose, we administered 90 μg PEDVS1 per 2 ml.

### Breeding, Vaccine Formulation, Immunization Schedule, and Experimental Timeline

Gilts were obtained from the Prairie Swine Centre (PSC; Saskatoon). Animal use was approved by the Animal Research Ethics Board, and all the interventions were carried out in accordance with the guidelines of the Canadian Council of Animal Care for Humane Animal Use. The timing of immunization is shown in Table 2. All gilts had their estrus cycles synchronized using oral progestin (Regu-Mate, Merck Animal Health, USA) for 14 days. When they showed signs of returning to estrus (2nd estrus), gilts referred to as the “i.u./i.m. group” (*n* = 4) were inseminated by conventional AI with a standard semen dose that included a 2 ml of NP vaccine injected into the 80 ml commercial semen bag before the insemination. The i.m. vaccine group was administered empty NPs (ARC4/7 encapsulated in PLGA NP, without PEDVS1 antigen or adjuvants) with live semen dose at 2nd estrus. Negative control gilts (*n* = 4) were administered only the live semen dose as per routine husbandry procedures. After 30 days, gilts from the i.m. group and the i.u./i.m. group received the vaccine (2 ml NP vaccine) on their shoulder muscle. Negative control gilts did not receive any mock i.m. vaccine. On approximately day 114 gestation, gilts were administered Planate (1 ml injection in the vulvar mucosa in the morning and afternoon; Merck Animal Health) to induce labor. Colostrum was collected on the day of farrowing and mature milk was collected on day 6. No cross-fostering of piglets took place, and litters were randomly culled to 10 piglets per gilt. Archived colostrum from gilts challenged with low dose PEDV plus alum was used as positive reference samples for neutralizing antibodies and passive protection.

**Table 2:** Schematic of vaccination schedule.

**Groups**	**Mucosal vaccine dose**	**Systemic vaccine dose**	**Total vaccine doses received**
i.u./i.m.	At breeding into uterus during 2nd estrus	i.m. vaccine administered 30 days after breeding and i.u. vaccination	2
i.m.	–	i.m. vaccine administered 30 days after breeding	1
Negative control	–	–	None

### Gilt Sampling

The timing of sampling is detailed in Table 3. Serum was collected on days 1, 30, 60, 100, and approximately day 125/126 for experimental gilts. Anti-PEDVS1 IgG and IgA antibody titers in serum and uterine mucosa were quantified over time (22). PBMCs from the gilts were isolated on day 100 and 6 days after piglets were challenged with PEDV to measure antigen-specific recall IFNγ cytokine expression. Although gilts were not directly challenged with the virus, they were exposed to the virus from their suckling piglets. Colostrum and mature milk samples were processed as detailed in Polewicz et al. (25) before being investigated for anti-PEDVS1 IgG and IgA antibodies and virus-neutralizing antibodies. Gilts were euthanized with 50 ml of Euthanyl (240 mg/ml; Bimeda-MTC Animal Health Inc., Cambridge, ON) when all piglets succumbed to the disease or at day 125/126 (up to 10 days after challenge). To obtain uterine mucosa from gilts (i.e., scrapings), a glass slide was gently applied to the uterine lumen and the mucosa was removed with a gentle scraping motion. The animals were exsanguinated immediately after birth to remove the vast majority of blood-derived antibodies in tissues. The uterine sampling was taken on the luminal side of the tissues and care was taken to avoid any blood when the tissues were scraped.

**Table 3:** Listing of animal numbers, assays, and groups.

**Groups**	**Gilts**	**Piglets**	**Sample collections**	**Assays**
**Overall immune response to vaccine**				
i.m. vaccinated gilts	4	NA	• Serum: Days 1, 30, 60, 100, and 125/126. • PBMCs: Days 100 and 125/126 • Uterine lining scraping and uterine luminal flush: Day 125/126 • Colostrum: Day of farrowing • Mature Milk: 6 days after farrowing	Antibody ELISA using serum, uterine lining scraping, uterine flush. Colostral antibody and neutralizing antibody ELISAs. IFNγ cytokine ELISA from PBMC's
i.u./i.m. vaccinated gilts	4	NA		
Negative control gilts	4	NA	Serum Days 1, 100, and 125/126 • PBMC's • Uterine lining scraping and uterine luminal flush: Day 125/126 • Colostrum: Day of farrowing • Mature Milk: 6 days after farrowing	
Positive control gilts	4	NA		Colostral neutralizing antibody ELISA
**Vaccine efficacy in suckling piglets challenged with PEDV at 3 days of age**				
From i.m. vaccinated gilts	4	40	Challenge with PEDV at 3 days of age	Piglets born, weighted at 3 days of age then challenged with PEDV. Clinical assessment and mortality scoring performed Piglets born, weighted at 3 days of age then challenged with PEDV. Clinical assessment and mortality scoring performed
From i.u./i.m. vaccinated gilts	4	40	Challenge with PEDV at 3 days of age	
From Negative control gilts	4	40	Challenge with PEDV at 3 days of age	

### Challenge With Infectious PEDV and Piglet Sampling

PEDV strain USA/Colorado/2013 (GenBank KF272920; GI:514483276) was obtained by the Diagnostic Virology Laboratory (NVLS, Ames, USA) and propagated as described previously (5).

Piglets were weighed at 3 days of age, then they were challenged with infectious PEDV at 3 days of age, but their dams were also indirectly exposed because they remained with their piglets during the infection period. Clinical analyses were performed on piglets daily. For the challenge, piglets were allowed to suckle their dams, and on the 3rd day of their life, they were orally challenged with live PEDV (3 × 10^2^ TCID_50_ per piglet) as detailed in Makadiya et al. (5). The piglets from the positive control gilts, from the vaccinated gilts and negative control gilts in the current trial, and the positive control gilts from a previous trial were challenged with the same lot of infectious PEDV. Clinical assessments of challenged piglets were performed as detailed in Choudhary et al. (22), with the exception that piglets that reached a cumulative score across depression, weight, and survival scores of 4 were euthanized.

### Immune Assays

Quantification of antigen-specific antibody ELISAs, colostral PEDVS1 IgG neutralizing antibody titers, IFNγ cytokine production from PBMCs in response to recall antigen, and quantitation of viral shedding in fecal samples were performed as detailed in Choudhary et al. (22).

### Statistics

All statistical analyses were carried out using GraphPad Prism 8 (GraphPad Software, San Diego, CA). For all ELISAs and clinical data sets, we tested data for normal distribution within each assay. If the data sets in an assay were normally distributed as determined using the Shapiro-Wilk test or the Kolmogorov-Smirnov test, we performed parametric analysis (unpaired *T*-test or Ordinary one-way ANOVA using Dunn's multiple comparisons, as appropriate). If the data were not normally distributed, we performed a non-parametric analysis (Mann-Whitney test or Kruskal-Wallis ANOVA using Dunn's multiple comparisons, as appropriate). The Log-rank (Mantel-Cox) test and the Gehan-Breslow-Wilcoxon test were used for the statistical comparison of survival curves. For the analysis of the PEDV clinical scores, differences between vaccinated and control animals were determined by unpaired *t*-test, using the Holm-Sidak correction for multiple comparisons.

## Results

### Systemic Humoral and Cell-Mediated Immune Response to Intramuscular and Joint Intrauterine/Intramuscular Vaccination

**Figure 1:**
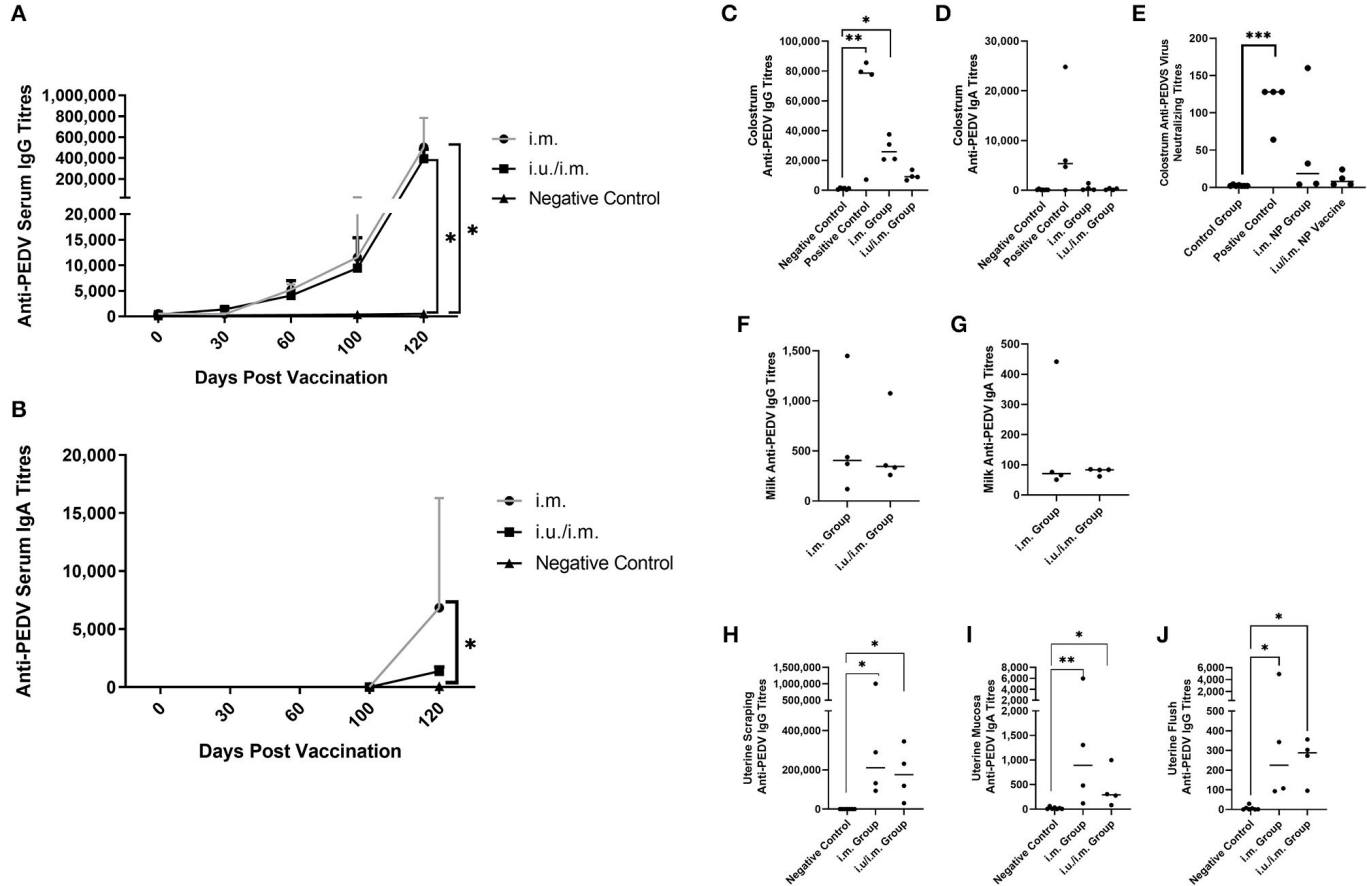
Serum and mucosal antibody titers from animals vaccinated through the intramuscular route with or without prior intrauterine vaccination. Serum IgG **(A)** and IgA **(B)** antibody titers were collected over time from gilts immunized with PEDVS1-NP 30 days after estrus (i.m. group) and from gilts immunized *via* the intrauterine route at breeding and followed by an i.m. vaccine 30 days later (i.u/i.m. group). Negative control gilts did not receive any vaccine. Positive control gilts were previously immunized with a live attenuated PEDVS1 vaccine and showed passive protection for piglets. IgG and IgA antibodies from colostrum [IgG and **(C)**; IgA and **(D)**], colostral virus neutralizing antibodies [IgG and **(E)**], and mature milk [IgG and **(F)**; IgA and **(G)**] were collected on the day of birth for colostrum and on day 6 for milk. At 9 days after farrowing, the uterine tissue was minced, and the mucosal antibodies were collected 48 h later to quantify uterine mucosal antibodies [IgG, **(H)**; IgA and **(I)**], and the uterine luminal antibodies were collected at the time of tissue harvest after 50 ml flush with PBS [IgG and **(J)**]. Data are presented as median and standard deviation. Statistical analysis was carried out by the Kruskal-Wallis test and Dunn's multiple comparisons test. Significant differences relative to the negative control gilt data are denoted by different asterisks (^*^p < 0.05, ^**^p < 0.01, ^***^p < 0.001).

We assessed the development of PEDVS1-specific antibody-mediated immunity over time (Figure 1A) in vaccines consisting of PEDVS1 antigen along with ARC4 and ARC7 adjuvants formulated as a PLGA NP. Serum was collected from gilts at estrus and then again at day 30, 60, and 100 gestation. Gilts vaccinated 30 days after estrus *via* the i.m. route with PEDVS1-NP (i.m. group) showed significant anti-PEDVS1 IgG (Figure 1A) and IgA (Figure 1B) in serum at day 100 gestation, 70 days after the i.m. immunization. The gilts immunized first *via* the i.u. route at estrus followed by an i.m. vaccination 30 days later (i.u./i.m.) showed comparable significant anti-PEDVS1 IgG titers indicating that the intrauterine vaccine, at least with this formulation and/or dose, did not act as a priming vaccine (Figures 1A,B). However, it is also important to note that the i.u. route of immunization did not trigger mucosal tolerance to the i.m. vaccine. When the piglets were infected with PEDV, the gilts were indirectly infected. Serum anti-PEDVS1 IgG and IgA antibodies were quantified, and we observed that the i.m. group responded with significantly more anti-PEDVS1 IgA relative to the negative control pigs (Figure 1B). Both vaccinated groups showed significantly elevated anti-PEDVS1 IgG, but the results were comparable (Figure 1A). We then quantified anti-PEDVS1 IgG (Figure 1C) and -IgA (Figure 1D) in colostrum, as well as colostral virus-neutralizing antibodies (Figure 1E), to discern how much antibody-mediated immunity could be passively transferred to the suckling piglets. Colostrum antibodies were compared to the negative control animals, as well as archived colostrum from “Positive control” gilts that were previously immunized with anti-PEDVS1 attenuated viral vaccine and whose colostrum protected piglets against infectious PEDV challenge (data not shown). The anti-PEDVS1 IgG antibody titers from the i.m. group were significantly elevated relative to the titers from negative control animals, yet the median titers were ~2-fold less than the titers in the colostrum from positive control gilts (Figure 1C). The i.u./i.m. vaccinated gilts showed colostral IgG titers that were not significantly different from the negative control gilts. Colostral anti-PEDVS1 IgA was relatively low across both vaccinated groups (Figure 1D). Neutralizing colostral IgG antibody titers were low across both vaccinated groups relative to the VN antibodies in the positive control colostrum, which were significantly elevated relative to the colostrum from the negative control gilts (Figure 1E). Mature milk was collected on day 6 after farrowing, and each vaccinated groups showed relatively low anti-PEDVS1 IgG (Median <500 titers) and IgA (Median <100 titers) titers (Figures 1F,G), respectively. Collectively, these results suggest that despite being collected several weeks post-immunization, significant colostral anti-PEDVS1 IgG titers were present in the colostrum from i.m vaccinated gilts and could be passively transferred to suckling piglets. However, the single i.u. vaccination failed to elevate colostral VN titers. The i.u./i.m. vaccinated gilts appeared to generate less colostral antibodies relative to the animals that received the i.m. dose alone. None of the immunization strategy promoted anti-PEDV IgA in colostrum or mature milk.

When the piglets were challenged with PEDV at 3 days of age, their respective gilts became indirectly exposed to the virus while tending their piglets. When we euthanized the gilts 9 days later, we obtained scrapings from the uterine horn, which were subjected to anti-PEDVS1 antibody ELISAs. We observed that both groups of vaccinated gilts produced significant uterine mucosal anti-PEDVS1 IgG (Figure 1H) and anti-PEDVS1 IgA (Figure 1I) titers relative to the titers from the negative control gilts. Further, the uterus was flushed before scraping, and the anti-PEDVS1 IgG antibodies were measured. The i.m. and i.u/i.m. vaccinated gilts had significantly higher anti-PEDVS1 IgG titers relative to the titers from the negative control gilts (Figure 1J). However, the antibody titers were low relative to the titers obtained from the scraped tissue, suggesting that the antibodies were not at a high concentration in the lumen but they were elevated in the mucosa itself.

### Cell-Mediated Immune Response

**Figure 2:**
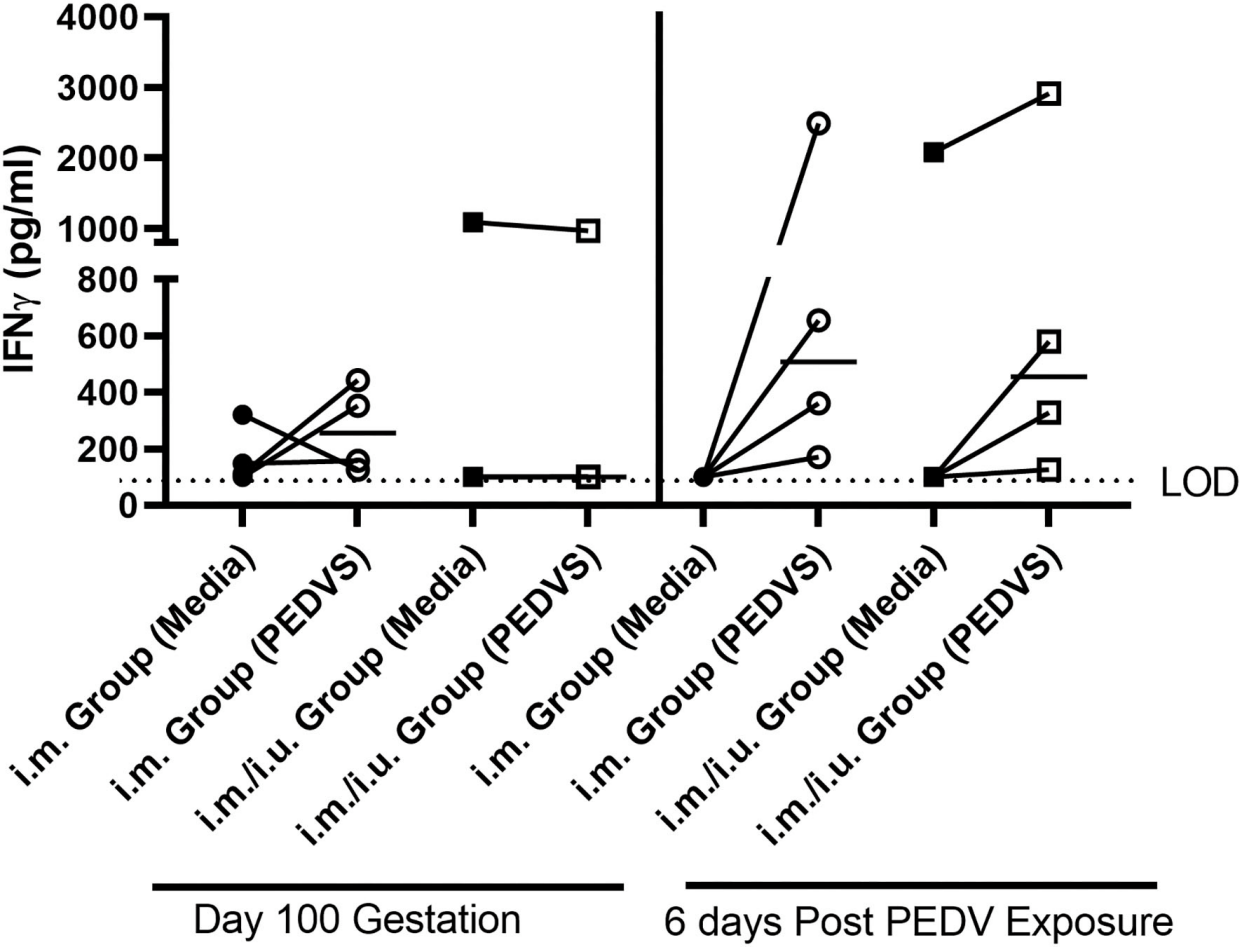
Cell-mediated immune responses quantified by gilts vaccinated with a single i.m vaccine or a i.m vaccine primed with an i.u. vaccine at breeding. IFNγ production was established using a cytokine ELISA kit. PBMCs were isolated on day 100 gestation and on day 9 after farrowing, which is 6 days post indirect exposure to PEDV. Each symbol represents one animal. ELISAs were conducted in Immulon 2 U plates and were read using the SpectraMax plus microplate reader and the limit of detection (LOD) is 100 pg/ml. Cytokine titers were graphed in GraphPad Prism 9. The median value is denoted by a horizontal bar and statistical comparisons made to unstimulated cells and cells stimulated with recombinant PEDVS1 protein using a Wilcoxon test.

To evaluate the potential of NP vaccines to elicit a systemic CMI response, PBMCs were obtained at day 100 gestation and on day 9 after farrowing, which is 6 days after the gilts were indirectly exposed to PEDV through their suckling and PEDV-challenged piglets. The PBMCs from the gilts were incubated with media or PEDVS1 protein for 2 days, followed by IFNγ ELISA analysis on the supernatants. The PEDVS1-specific IFNγ response from both groups of vaccinated gilts did not promote a recall response at day 100 gestation. After they were indirectly exposed to the virus through piglets for 6 days, PBMCs from the gilts showed an increase in IFNγ recall response, although the data did not meet the standards of statistical significance (*P* < 0.125). These data suggest that at least at this dose, the i.m. and i.u./i.m. vaccine did not induce a robust CMI recall response (Figure 2).

### Live Births and Growth Kinetics of Piglets Born to Vaccinated and Control Gilts

**Figure 3:**
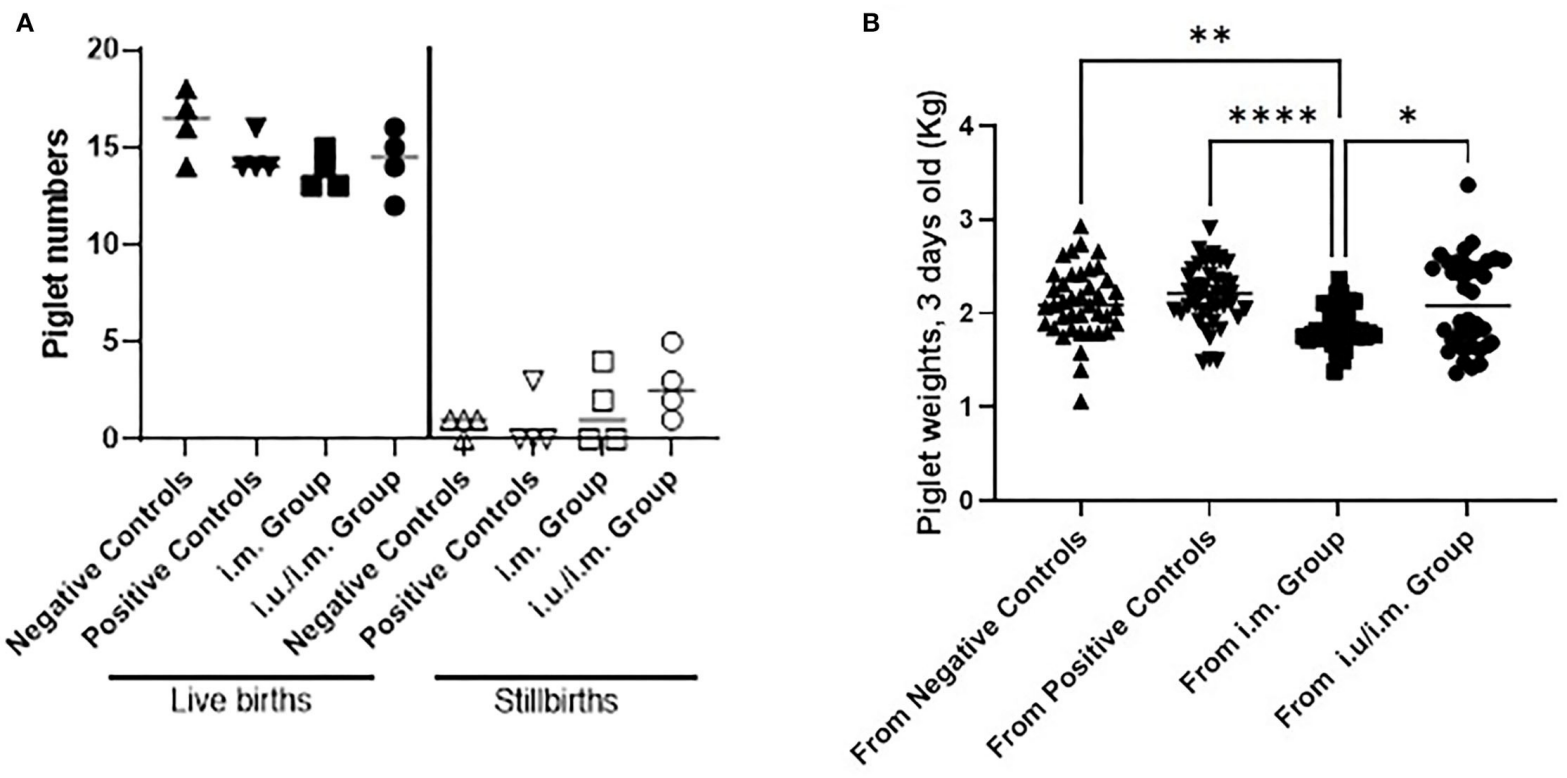
Births and weights of piglets born to control gilts and gilts vaccinated through the intramuscular route with or without prior intrauterine vaccination. Gilts were immunized with PEDVS nanoparticle 30 days after estrus (i.m. group) or they were immunized *via* the intrauterine route at breeding and followed by an i.m. vaccine 30 days later (i.u./i.m. group). Negative control gilts did not receive any vaccine. Positive control gilts were previously immunized with a live attenuated PEDV vaccine and showed passive protection for piglets. **(A)** The number of live births per litter and the number of stillbirths per litter were recorded. Each symbol represents the number of piglets per gilt. The mean value is denoted by a horizontal bar with standard deviation indicated as error bars. **(B)** Piglets were weighed at 3 days of age (kg). Data are shown as the birth weights from all piglets born to each gilt with median weights shown by a horizontal line. Statistical comparisons were made between the control and vaccinated animals **(A)** and between all groups in **(B)** using the Kruskal-Wallis test and Dunn's multiple comparisons test. Significant differences are denoted by different asterisks (*p < 0.05, **p < 0.01, ****p < 0.0001).

We measured the number of live piglets born and stillbirths for both sets of vaccinated gilts relative to data from negative control and positive control gilts (Figure 3A). We observed no significant differences between the number of live births or stillborns delivered by the control gilts and vaccinated gilts, suggesting that i.m. vaccination with an NP with or without a priming vaccine at breeding did not negatively affect fertility. We next measured the weight of piglets at 3 days of age, and we observed that the weights of the piglets born from i.m. vaccinated gilts were significantly smaller compared to the other groups but that there are large amounts of variation in weights in piglets across gilts (Figure 3B). The number of gilts per group would need to be increased to subjectively be assured that the i.m. vaccination alone was impacting piglet birth weights.

### Assessment of Passive Protection for Suckling Piglets Born From Vaccinated Gilts

#### PEDV Infection at 3 Days of Age

**Figure 4:**
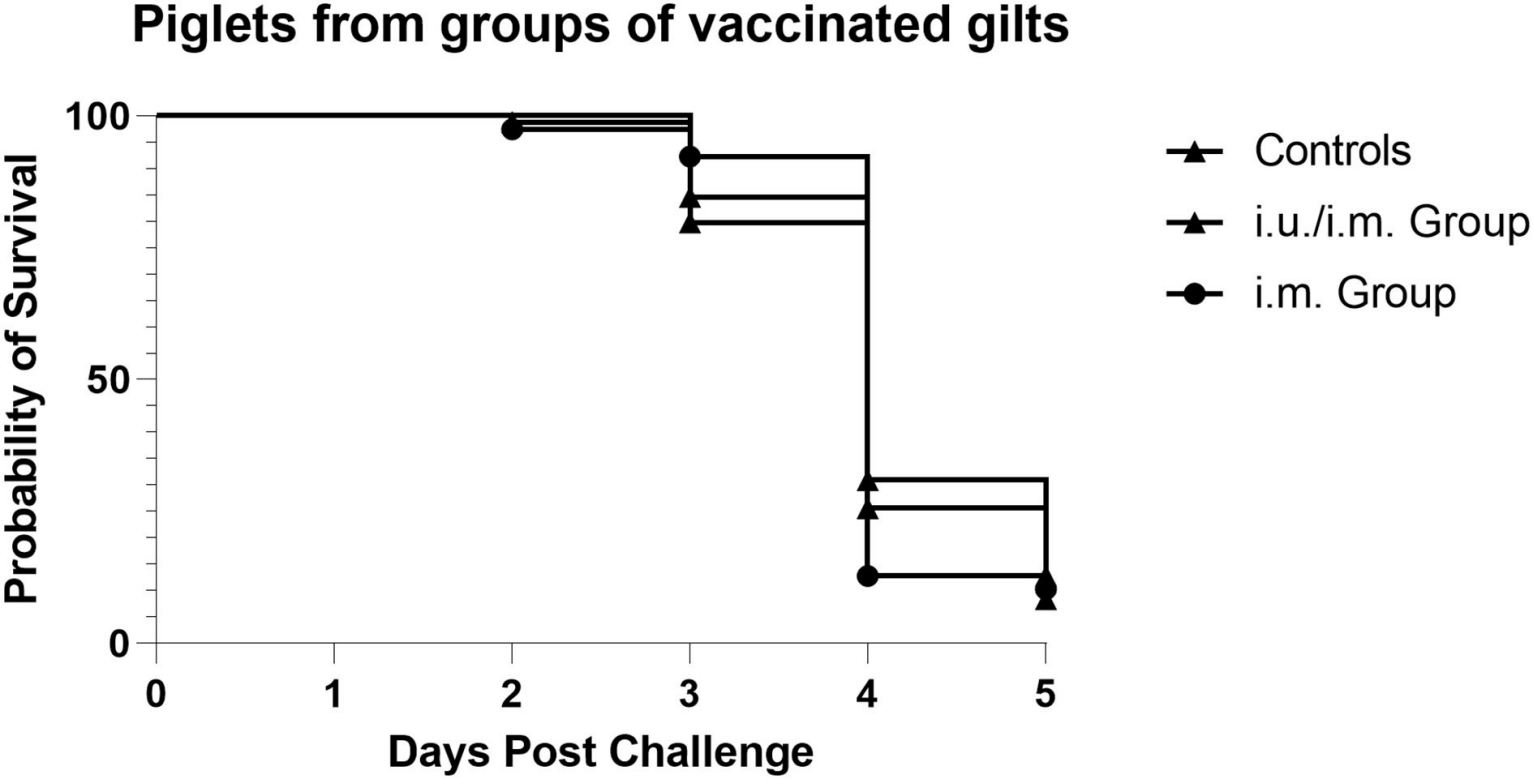
Survival of piglets when challenged with infectious PEDV 3 days after birth. The majority of the piglets born from negative control gilts and piglets born from i.m. vaccinated gilts and i.u./i.m. vaccinated gilts were euthanized on day 4 when their cumulative depression and weight loss scores reached a score of 4.

Vaccinated gilts remained in relatively tight synchronicity for farrowing and were moved to BSL3 animal containment at ~100 days gestation. Piglets were orally challenged with infectious PEDV at 3 days of age (Day 0). Piglet weight loss scores and depression scores were tabulated each day until 9 days of age with the percentage being compared to the day of challenge for each group equaling 100% on day 0 (Table 2). The grading score was changed from a score of > 3 per criteria to a grading score of 4 or higher cumulative average, so comparisons across trials cannot be performed (Table 4). However, the majority of pigs from the vaccinated groups were euthanized on day 4, which suggests that they were not sufficiently protected against the infectious challenge (Figure 4). These results suggest that a single i.m. immunization or an i.u./i.m. immunization with PEDVS1 ARC4/7 PLGA NP did not provide passive protection to suckling offspring. These data show agreement with Langel et al. (1), which showed that piglet survival positively correlated with high PEDVS1 IgA antibodies and virus-neutralizing antibody in milk (1).

**Table 4:** Clinical assessments of piglets after viral challenge.

**Criteria**	**Scores**	**Day 1**		**Day 2**		**Day 3**		**Day 4**		**Day 5**		**Day 6**		**Day 7**	
**Groups**		**i.m**.	**i.u./ i.m**.	**i.m**	**i.u./ i.m**.	**i.m**	**i.u./ i.m**.	**i.m**	**i.u./ i.m**.	**i.m**	**i.u./ i.m**.	**i.m**	**i.u./ i.m**.	**i.m**	**i.u./ i.m**.
Weight Scores	0	34	39	1	5		2		1	2		2			1
	1	5	1	24	23	3	10	3	5		3		2		1
	2	1		13	8	11	10		2		2		1		
	3			1	4	23	12		3		1				
	4														
Dead/euthanized				1		2	6	34	23	1	5		3		
Depression scores	0		40							2		2			2
	1	30		29	29	2	25						2		
	2	9		10	11	31	4	3	10		6		1		
	3	1		1		4	6		1						
	4														
	5														

## Discussion

Mucosal vaccines induce both systemic and mucosal immunity and have the potential to control pathogens at their point of entry. Systemic vaccines are generally not recognized as inducing immunity at mucosal sites, which is where an estimated 90% of all infectious pathogens invade. Therefore, there has been a push toward the development of mucosal vaccines that can protect systemic and mucosal sites. Furthermore, mucosal immunization and non-lethal challenge of gilts may lead to adequate colostral and milk antibodies to passively protect piglets against infections challenge. For example, Langel et al. (1) showed non-lethal PEDV infection of gilts in the second trimester resulted in significantly higher levels of circulating PEDVS1 IgA and IgG antibodies and antibody-secreting cells and PED virus neutralizing (VN) antibodies post-PEDV infection, coinciding with 100% survival rate of their PEDV-challenged piglets compared with 87.2, 55.%, and 5.7% for first, third, and mock litters, respectively (1).

Mucosal tolerance is a major immunological process that occurs continuously at all mucosal sites designed to prevent local and peripheral overreaction to innocuous antigens (26, 27). Locally produced sIgA or sIgM bind antigens to mask their epitopes, thus preventing an inflammatory response, while also preventing microbial colonization and penetration of the gut wall (28). However, there may be biological consequences associated with prior non-infectious exposure to pathogens, which can impact response to vaccines. If an animal first encounters an antigen *via* a mucosal route, such as orally or in the urogenital tract with fomites, re-exposure to the antigen by a systemic route may result in suppression of immunity rather than induction of immunity, which is not ideal for vaccination. Factors influencing whether mucosal tolerance or immunity is induced in response to antigen include how antigens are presented to lymphocytes, the host's immunological maturity at time of exposure, the timing and the frequency of exposure, and the nature of the antigen (27, 29–33). We showed that a single exposure to a soluble i.u. vaccine consisting of 800 μg protein antigen with the VIDO triple adjuvant [TriAdj: 400 μg Poly I:C (polyinosinic:polycytidylic acid), 800 μg HDP (host defense peptide) and 400 μg PCEP (polyphophazene)] or binary ethylenimine inactivated (BEI) PPV (Porcine parovirus) virus formulated with TriAdj was not sufficient to promote strong antibody-mediated immunity in serum or mucosal tissues (34). In contrast, we also observed that 3 × i.u. immunization with soluble PEDVS1 plus TriAdj led to significant anti-PEDVS1 in serum, uterine mucosal, and colostrum response (22). In fact, colostral neutralizing antibodies were significantly induced relative to control gilts, but the titers were not sufficient to protect against infectious PEDV to suckling piglet's response (22). However, the current trial showed that an i.u. NP vaccine coupled with an i.m. NP vaccine induced an ~10 × higher serum anti-PEDVS1 IgG response relative to the thrice immunization of the uterus with the soluble PEDVS1 vaccine in pigs (22). The colostral anti-PEDV IgG titers, but not the IgA titers, were significantly elevated, suggesting that an NP formulation may be a better immunization formulation and should be studied further.

We previously investigated the immunization into the uterus followed by i.m. immunization in the pig triggers mucosal tolerance; however, the trial setup had important differences which could impact the interpretation of the results. Previous experiments in our laboratory have shown that a single i.u. immunization with soluble subunit protein with TriAdj followed up with 2 i.m. immunizations 3 weeks apart (22). The anti-FliC serum IgG response was very low (~400 anti-FliC IgG titers), such that we were not clear whether tolerance may have been induced. However, the i.u./2 × i.m. FliC-soluble vaccine regime did trigger significant and relatively high IFNγ titers suggesting induction of CMI immunity. However, the comparisons we made were to gilts immunized three times into the uterus with PEDV-soluble vaccine. The use of different antigens makes it difficult to establish whether differences in the measured immune response were due to the route or simply the immunogenicity of the antigen. Therefore, in the current trial, we wanted to establish whether an NP-based vaccine could impact the immune tolerance, and we used the same antigen in both groups of vaccinated gilts. We show that the anti-PEDVS1 titers in serum from the gilts immunized with the i.m vaccine or i.u./i.m. both triggered ~10,000 anti-PEDVS IgG titers after 70 or 100 days post respective vaccination. In contrast, the PEDVS1-specific CMI response in the current trial was low at day 100 gestation regardless of whether the i.m. injection was preceded by an i.u. immunization or not. However, when the gilts became indirectly exposed to the virus from the challenged suckling pigs, we observed a trend toward the induction of PEDVS1-specific CMI response. Clearly, the NP formulation did not promote mucosal tolerance. Results also make it clear that repeated i.u. immunization alone with a soluble vaccine (22) or with i.m. injection NP did not significantly promote protective passive immunity. More experimentation is required with multiple doses (i.e., at each breeding cycle) with increased doses of antigen and/or different combinations of adjuvants are required before a suitable i.u. vaccine will protect piglets against neonatal diseases and gilts/sows against reproductive diseases. Vaccine strategies may need to be different depending on whether one is targeting a reproductive disease, such as PRRSV or PPV, or an enteric disease, such as PEDV. For example, i.u. priming followed by an i.m. boost may be necessary to protect against PRRSV as IgA antibody-secreting cells trafficking from the uterus to mature milk is not well-documented. However, intestinal priming/infection with PEDV is demonstrated to produce robust anti-PEDV antibodies in mature milk leading to piglet protection (1), suggesting that priming of the intestine, not the uterus, may impact antibody levels in colostrum and milk. Others showed that three i.m. immunizations with PEDVS1 plus TriAdj administered starting 46 days before farrowing led to significant serum antibody titers and VN titers to protect piglets against infectious PEDV (5). Different strategies may be necessary to promote effective mucosal immunization, depending on the disease.

PLGA NPs act as potential delivery systems for vaccine formulations. Modification of physical properties of PLGA could shift the delivery of encapsulated antigens to either cytoplasm (for MHC I presentation and CD8+ T cell activation) or the endosome (for MHC II presentation and CD4+ T cell activation). According to previous studies, cytoplasmic delivery of PLGA content is affected by differences in the molecular weight of PLGA (35). Mucosal delivery of PLGA NPs-based vaccine has been shown to elicit the immune response required to induce T-cell response and clear viremia in a pig model. Intranasal delivery of PLGA NP-entrapped sonicated PRRSV antigens from VR2332 strain (Nano-KAg) was reported to significantly increase the virus-neutralizing titers in the lungs compared to both unvaccinated and killed vaccine vaccinated pigs (36). The lung homogenate and sera of Nano-KAg vaccinated pigs had higher levels of IFN-γ and lower levels of TGF-β than the control groups and could complete clearance of viremia in just 2 weeks. In addition, inactivated influenza virus antigens encapsulated in PLGA-NPs reduced the clinical disease and induced a cross-protective cell-mediated immune response in a pig model (37). Moreover, Norovirus P particle containing the extracellular domain of matrix protein 2 chimera and highly conserved H1N1 peptides from pandemic 2009 and classical human influenza virus were encapsulated within PLGA-NPs (38). They were administered with or without the adjuvant *Mycobacterium vaccae* whole cell lysate. Pigs were administered with the vaccine intranasally as a mist, and the vaccine induced the virus-specific T-cell response in the lungs and reduced the virus load in the airway of pigs upon challenge. Thus, PLGA-NPs have been shown to be effective as a vaccine delivery vehicle to mucosal sites in pigs.

In a mouse trial, we showed that OVA with ARC4/7 adjuvants formulated in polymeric PLGA NPs triggered robust antibody-mediated immunity in serum and IFNγ CMI and significant lymphocyte proliferation relative to the mice immunized with the unadjuvanted vaccine (10). Others have shown the MPLA combined with *Streptococcus suis* proteins provides immunological protection in pigs (17). Pigs have also been shown to be responsive to MDP (18), so it is reasonable to assess whether these adjuvants will trigger an innate immune response in pigs. Our results showed that the NP vaccine failed to trigger a CMI response, although when the gilts were indirectly exposed to the virus through their piglets, the T cells appeared to show a trend toward induction of antigen-specific CMI.

The purpose of this study was to evaluate whether a single i.m. vaccine with PLGA NP encapsulating a PEDVS1 antigen and adjuvants could trigger antigen-specific mucosal and systemic immunity in pigs resulting in passive protection for piglets. Results show that the NP vaccine triggered significant anti-PEDVS1 IgG and IgA in serum and uterine tissue and IgG in colostrum but not mature milk. Despite the significant induction of immunity in the gilt, the concentration of virus-neutralizing antibodies the single administration of the i.m. NP vaccine, at least with this formulation, failed to generate sufficient passive immunity to protect against infectious disease. The protective immune response should be assessed with an antigen for PRRSV or PPV to see if the i.m. NP vaccine alone or primed with an i.u. vaccine may contribute to protective immunity in the gilts against reproductive diseases. Immunization of gilts at breeding alone or with a second systemic immunization would be viewed positively by the pig industry because it will reduce person-power (combining immunization with breeding) while still triggering protective immunity alone or after being coupled with a single needle-based injection.

## Data Availability Statement

The datasets presented in this study can be accessed by contacting the corresponding author: heather.wilson@usask.ca.

## Ethics Statement

The animal study was reviewed and approved by University of Saskatchewan Animal Research Ethics Board.

## Author Contributions

PC and SN with NB performed the experiments and data analyses. AH, AK, and RM were responsible for providing the expertise for NP formulation and in the actual formulation and quantification. DY from the Alberta Research Chemicals Inc. provided the ARC4 /7 adjuvant expertise and assisted in the experimental design. HW assisted in the experimental design and data analysis and wrote the first draft of the manuscript. All authors contributed to the article and approved the submitted version.

## Funding

VIDO receives operational funding from the Government of Saskatchewan through Innovation Saskatchewan and the Ministry of Agriculture and from the Canada Foundation for Innovation through the Major Science Initiatives for its CL3 facility. The funding for this research was provided by the Saskatchewan Agriculture Development Fund and the Saskatchewan Ministry of Agriculture and the Canada-Saskatchewan Growing Forward 2 b-lateral agreement.

## Conflict of Interest

DY was employed by Alberta Research Chemicals Inc. The remaining authors declare that the research was conducted in the absence of any commercial or financial relationships that could be construed as a potential conflict of interest.

## Publisher's note

All claims expressed in this article are solely those of the authors and do not necessarily represent those of their affiliated organizations, or those of the publisher, the editors and the reviewers. Any product that may be evaluated in this article, or claim that may be made by its manufacturer, is not guaranteed or endorsed by the publisher.

## Supplementary material

Supplementary Figure 1 Serum antibody titers from animals vaccinated through the intramuscular route with or without prior intrauterine vaccination. Serum IgG **(A)** and IgA **(B)** antibody titers were collected 120 days post-breeding which would be 120 days post i.u. immunization and 90 days post i.m. immunization with PEDVS1 Nanoparticles. Negative control gilts did not receive any vaccine. Data are presented as median and standard deviation. Statistical analysis was carried out by the Kruskal-Wallis test and Dunn's multiple comparisons test. Significant differences relative to the negative control gilt data are denoted by different asterisks (^*^*p* < 0.05, ^**^*p* < 0.01).
