# Supplementary material for: A Single-Dose Intramuscular Nanoparticle Vaccine With or Without Prior Intrauterine Priming Triggers Specific Uterine and Colostral Mucosal Antibodies and Systemic Immunity in Gilts but Not Passive Protection for Suckling Piglets
Source: Front Vet Sci. 2022 Aug 3;9:931232. doi: 10.3389/fvets.2022.931232 (PMC9383261; doi:10.3389/fvets.2022.931232)
Supplement: Supplementary Figure 1 — Serum antibody titers from animals vaccinated through the intramuscular route with or without prior intrauterine vaccination. Serum IgG (A) and IgA (B) antibody titers were collected 120 days post-breeding which would be 120 days post i.u. immunization and 90 days post i.m. immunization with PEDVS1 Nanoparticles. Negative control gilts did not receive any vaccine. Data are presented as median and standard deviation. Statistical analysis was carried out by the Kruskal-Wallis test and Dunn's multiple comparisons test. Significant differences relative to the negative control gilt data are denoted by different asterisks (*p < 0.05, **p < 0.01). [file Image_1.pdf]

Supplementary Figure1:

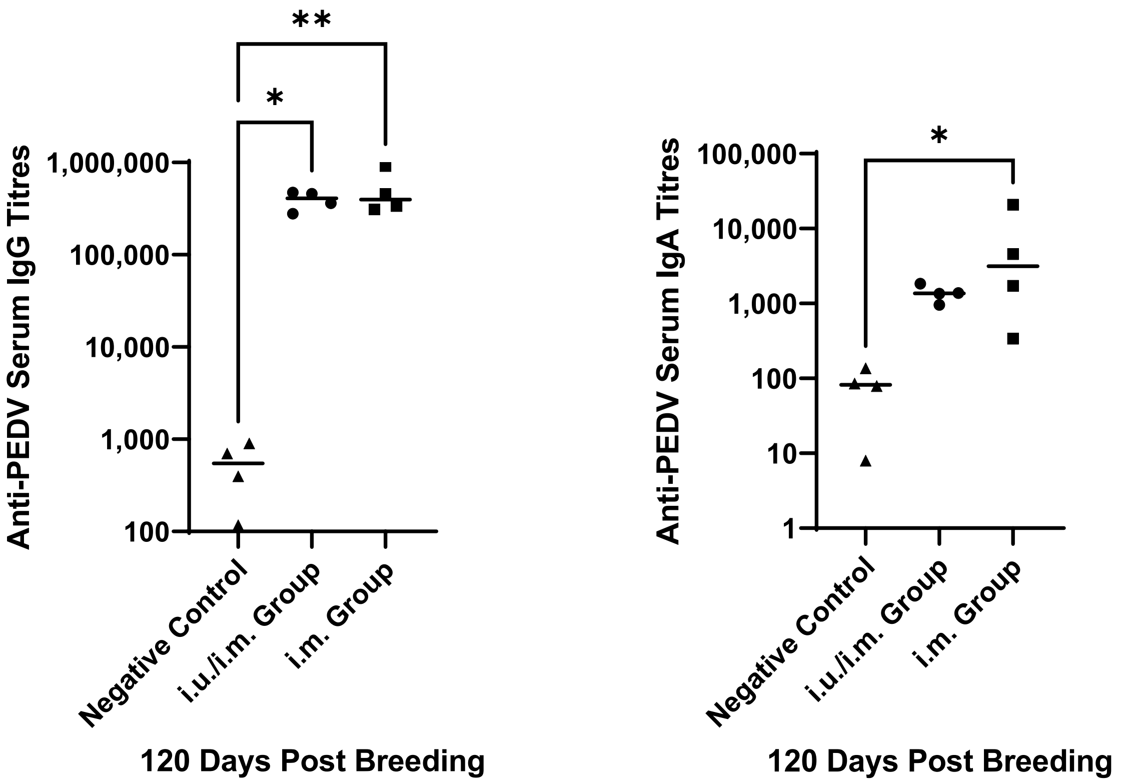

Supplementary Figure 1: Serum antibody titers from animals vaccinated through the intramuscular route with or without prior intrauterine vaccination. Serum IgG (A) and IgA (B) antibody titres were collected 120 days post breeding which would be 120 days post i.u. immunization and 90 days post i.m. immunization with PEDVS Nanoparticles. Negative control gilts did not receive any vaccine. Data are presented as median and standard deviation. Statistical analysis carried out by Kruskal-Wallis test and Dunns multiple comparisons test. Significant differences relative to the negative control gilt data are denoted by differing asterisks (* $p<0.05$, ** $p<0.01$).
